# Embryos of an Antarctic zooplankton require anoxia for dormancy, are permeable to lipophilic chemicals, and reside in sediments containing PCBs

**DOI:** 10.1038/s41598-018-34689-w

**Published:** 2018-11-02

**Authors:** Katherine A. Reed, Hyun Park, Sung Gu Lee, Wonseok Lee, Sang-Hwan Lee, Jason M. Bleau, Taylor N. M. Munden, Joseph A. Covi

**Affiliations:** 1 The University of North Carolina at Wilmington, Department of Biology and Marine Biology, 601 S College rd., Wilmington, NC 28403 USA; 2 Unit of Polar Genomics, Korea Polar Research Institute (KOPRI), 26 Songdomirae-ro, Yeonsu-gu, Incheon 21990 Korea; 3 National Institute of Environmental Research, Incheon, 22689 Korea; 4 Mine Reclamation Technology Center, Korea Mine Reclamation Corporation, Wonjusi, Gangwando 26464 Korea

## Abstract

Zooplankton in Antarctic maritime lakes face challenges imposed by anthropogenic chemicals. Studies on temperate species suggest that lipophilic chemicals will accumulate in dormant embryos of Antarctic zooplankton and decrease hatching success, thereby threatening centuries of accumulated genetic diversity that would increase population resilience in the face of climate change. We evaluated the potential for lakes to act as sinks for legacy pollutants in the maritime Antarctic by testing sediments for polychlorinated biphenyls (PCBs) previously identified in soil, flora and fauna of lake catchments. Direct tests of embryo permeability to chemicals are confounded by potential adhesion of chemicals to the embryo surface and limited biomass available. Therefore, in order to assess the potential for lipophilic chemicals to penetrate and passively accumulate in dormant embryos of Antarctic lacustrine zooplankton, we evaluated the effect of anoxia on post-diapause development in the calanoid copepod, *Boeckella poppei*, and then used chemical anoxia induced by rotenone as a reporter for permeability of these embryos to moderately lipophilic chemicals. The data presented demonstrate that embryos of *B*. *poppei* from Antarctic lake sediments will passively accumulate moderately lipophilic chemicals while lying dormant in anoxic sediments. Implications for legacy POPs in sediments of Antarctic maritime lakes are discussed.

## Introduction

To maintain the population in harsh and variable environments, inland and coastal zooplankton on all seven continents produce embryos capable of surviving decades to centuries in a cryptobiotic state^1–4^, and this life-history strategy makes them uniquely susceptible to anthropogenic chemical influence. Embryos of the calanoid copepod, *B*. *poppei*, were recently isolated from 196 year-old sediments in freshwater lakes on King George Island, South Shetland Islands, Antarctica^5^. The extreme age of these embryos suggests that “cryptobiotic” dormancy plays important ecological and evolutionary roles for this species. Evolutionary biologists and ecologists have clearly demonstrated the importance of similar cryptobiotic “egg banks” to lacustrine communities elsewhere^1,2,6–8^. Unfortunately, anthropogenic influence on these embryonic storehouses of genetic diversity remain largely unstudied^9^. We propose that development after dormancy is the key stage where the impacts of anthropogenic chemicals will manifest in species like *B*. *poppei*, because zooplankton embryos are preloaded *in utero* with chemicals that bioaccumulate in the food web^10,11^ and sit for years in bottom sediments where passive accumulation of chemicals continues^9,12^. Lipid stores are mobilized to support early zooplankton development^13–15^, and the impacts of lipophilic chemicals manifest when they are released as lipid stores are used^16–18^. Given this, it is surprising that little research has been conducted on the impacts of anthropogenic chemicals on egg bank viability.

Persistent organic pollutants (POPs) are broadly distributed in remote cold environments by biological vectors and atmospheric transport combined with cold precipitation, but the deposition of these compounds in Antarctic maritime lakes has been largely ignored. Atmospheric transport brings anthropogenic chemicals to remote sites in the Arctic, including a diverse array of organochlorines and polycyclic aromatic hydrocarbons (PAHs)^19–21^. Similar atmospheric deposition occurs in remote regions of the Himalayas^22^ and the Antarctic^23–25^. Multiple studies document the presence of PAHs, polybrominated diphenyl ethers (PBDE) and numerous organochlorines in Antarctic terrestrial soil, mosses, marine sediment, seawater, benthic marine organisms, fish and seabirds^26–32^, and cycling of volatile POPs between aquatic, terrestrial, snow and ice deposition sites is continual^33^. Because seabirds act as vectors that transport POPs to terrestrial environments around lakes^26,34,35^, and meltwater containing POPs enters lakes during the austral summer, it is reasonable to predict that POPs are present in maritime lakes which support relatively isolated populations of zooplankton. Despite these facts, neither lakes nor maritime lacustrine zooplankton are discussed in reviews of chemical pollution in Antarctica.

Two distinct states of dormancy are recognized in the embryos of zooplankton, diapause and quiescence^36^. Diapause is induced as part of a developmental program, and must be broken by one or more environmental cues for development to continue^36–38^. By contrast, quiescence is environmentally induced and prevents the embryo from developing under non-permissive conditions, such as anoxia^36,37,39^. Metabolic suppression can be so complete during quiescence that heat production and substrate utilization become nearly undetectable within days of initiating dormancy^40,41^. When permissive environmental conditions are restored, quiescent embryos resurrect from their dormant state and rapidly emerge as motile larvae^2,42,43^. Individuals in the diapause state may enter a quiescent state if adverse conditions are encountered after diapause is broken, and embryos found in natural sediments are often a mix of these two distinct states.

Lipophilic chemicals readily pass through arthropod cuticles^44^, including embryonic cuticles of cladocerans, copepods and anostracans^9,12,45–48^. Invertebrate embryos, including those of calanoid copepods^14^, also possess rich intracellular lipid stores where lipophilic chemicals could accumulate by passive partitioning. Importantly, dormant embryos may also lack the metabolic capacity required to breakdown or export such toxicants, and these embryos often lie in sediments devoid of light and oxygen that would otherwise facilitate chemical breakdown by oxidative processes. Thus, unless they possess a specialized barrier to lipophilic compounds, dormant embryos of *B*. *poppei* will passively bioaccumulate lipophilic compounds. Testing this hypothesis is complicated by the fact that dormant *B*. *poppei* in Antarctic lake sediments are impossible to obtain in amounts sufficient for chemical analyses, and, even if they were available, such tests would not differentiate between adhesion of chemicals to the outer surface of the embryo and intra-embryonic accumulation. To further complicate tests of embryo permeability to POPs, it would be necessary to maintain the dormant state in the presence of a chemical that would not adversely impact embryo viability during dormancy, and there are no published data to base such a study on. In the present study, we circumvent these problems by using a biological reporter assay. We first document the response to anoxia in embryos of *B*. *poppei*, and then use chemical anoxia induced by rotenone to evaluate the permeability of these embryos to moderately lipophilic compounds. The well characterized model zooplankton, *Artemia franciscana*, is used as an oxygen scavenger and to validate the methods employed for testing the response of *B*. *poppei* to anoxia. The results demonstrate that embryos of *B*. *poppei* found in Antarctic lake sediments depend on anoxia to maintain the dormant population, and that the protective cyst wall of these embryos is permeable to moderately lipophilic chemicals.

## Results

### Early post-diapause development of *B*. *poppei*

**Figure 1:**
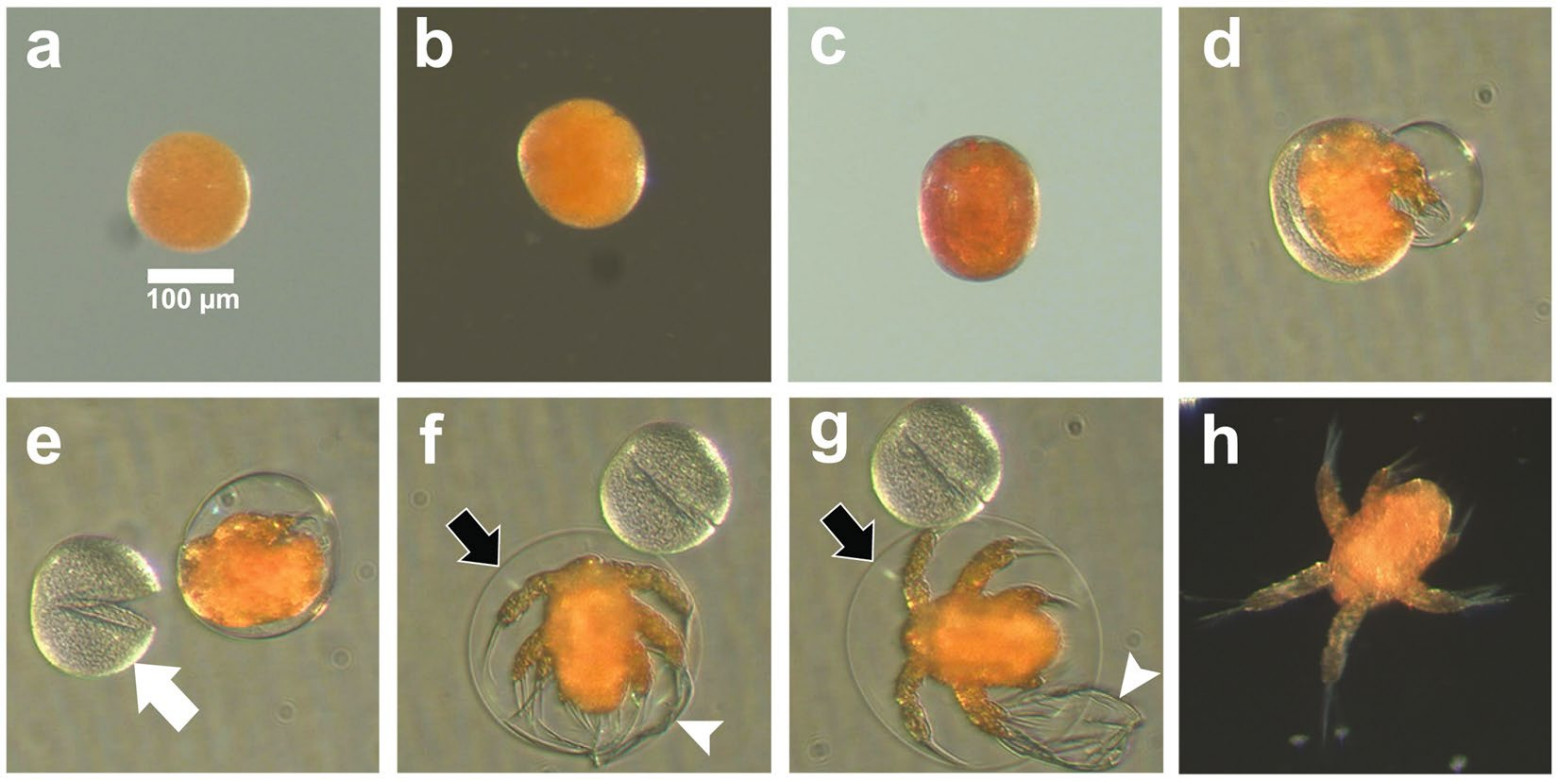
Post-diapause development, emergence, and hatching as observed by light microscopy in live *B*. *poppei* isolated from sediment of lake on King George Island, Antarctica. (**a**) Early stage of development (ED Embryo). (**b**) Intermediate stage of development (ID Embryo). (**c**) Pre-nauplius. (**d**) Emergent pre-nauplius. (**e**) Outer wall (white arrow) of cyst shed. (**f**) Inner cyst wall (white arrowhead) shed as the hatching membrane (black arrow) expands. (**g**) Hatching membrane fully expanded; nauplius begins burst swimming (Supplementary Video S1). (**h**) Free-swimming nauplius larva.

Both developmental patterning of the embryo and molting events were easily observed under a dissecting microscope at 100X magnification (Fig. 1). The majority of embryos of the copepod, *B*. *poppei*, isolated from sediments of lakes on King George Island, Antarctica, retained red or orange coloration and lacked bilateral symmetry, anterior/posterior axis and segmentation visible at 100X magnification (Fig. 1a). These embryos were presumed to be in diapause, because they did not develop, hatch or deteriorate when isolated from the sediment and incubated at 4 °C under aerobic conditions with constant light or a 19:5 light:dark cycle to mimic conditions of the austral summer. Live embryos in the diapause state could not be differentiated from embryos in an early stage of post-diapause development with light microscopy, so these were grouped together and designated as the Early Development (ED) embryonic stage. Development in progress first became apparent when bilaterally symmetrical spaces appeared between the cyst wall and the inner embryo mass at one end of the embryo in a stage designated as the Intermediate Development (ID) embryo (Fig. 1b). There was some variability in developmental timing from individual to individual. Generally, within 48 h of reaching the ID embryo stage, the individual progressed to the pre-nauplius stage, which was characterized by an oval shape with well-defined bilateral symmetry and body axis formation (Fig. 1c). Twitching of muscles was visible late in the pre-nauplius stage. Hatching of the nauplius larva started with the rupture of the outer cyst wall (Fig. 1d; Video S1) and complete emergence of the nauplius inside of a flexible multi-layer structure that is shed in two stages as the space surrounding the nauplius expands and a thin hatching membrane is stretched (Fig. 1e-g; Video S1). The nauplius swam in rapid burst patterns until the thin hatching membrane ruptured, releasing the free-swimming nauplius (Fig. 1h; Video S1).

### Evaluation of salinity and light impacts on hatching of *B*. *poppei*

**Figure 2:**
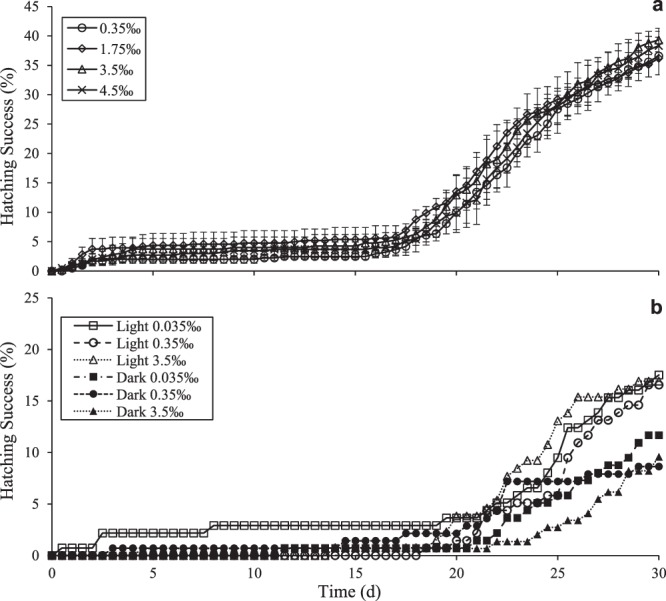
Effect of salinity and light on hatching success of *B*. *poppei*. (**a**) Lack of sensitivity to salinity between 0.35‰ and 4.5‰ ASW. Hatching success was evaluated as the percent of all individuals reaching the free-swimming nauplius stage with T = 0 as day of isolation of red embryos from sediment, and initiation of plate culturing. One way ANOVA demonstrated that there were no significant differences among treatment salinities when 1 d, 5 d, 10 d, 15 d, 20 d, 25 d or 30 d were considered as endpoints (n = 3, α = 0.05). (**b**) Hatching success was greater under light than in total darkness.

Progression from the ED stage of development to the fully formed nauplius requires less than 24 h in some individuals, but an extended exposure to light under aerobic conditions is required for most embryos to begin post-diapause development (Fig. 2). When ED embryos are incubated at 2 °C–4 °C under constant light in media equilibrated with room air, the first free-swimming nauplii were observed after 24 h (Fig. 2). While a small subset of embryos reached the nauplius stage within three days of incubation under these conditions, the second larger hatching event did not begin until two weeks after the initial small hatching event was completed (Fig. 2). Variation of salinity between 0.035‰ and 4.5‰ by dilution of ASW had no significant effect on hatching success at any time point (one way ANOVA, p > 0.45) (Fig. 2). Evaporation from the culture plates was less than 0.5% (5 µl) per week, so salinity change over time was negligible.

Restriction of light exposure by only exposing embryos to light during daily observation did not change the timing of the mass hatching event, but did decrease the total number of individuals that hatched within 30 d (Fig. 2b). It is important to note that, due to limitations in the number of embryos available for the study, it was not possible to terminate experiments after a single exposure to light during daily microscopy. As a consequence, all embryos were exposed to 5030 ± 100 lux for approximately 5 min each day under the microscope for the first two weeks of each experiment. By the end of the 30 d experiments, the time of exposure each day increased to 25 min, because of the need to evaluate some embryos in a state of decay.

### Evaluation of PCB content in Antarctic lake sediments containing *B. poppei*

The most common PCB homologues in sediments samples from two lakes on Barton Peninsula, King George Island, Antarctica, that contain *B*. *poppei* are Di, Tri and Tetra-PCBs (Table 1). Penta and Hexa-PCBs are also found in some samples, but at concentrations less than 260 µg g^−1^ sediment. The total PCB load was approximately 1 ng g^−1^ sediment, and the Di and Tri-PCBs accounted for over 2/3 of that value.

**Table 1:** PCBs in bottom sediment of two lakes with populations of *B*. *poppei* on Barton Peninsula, King George Island, Antarctica (*ng PCB g*^*−1*^ *sediment*).

PCB Homologue	Lake 1				Lake 2			
	Site 1	Site 2	Site 3	Mean (SEM)	Site 1	Site 2	Site 3	Mean (SEM)
Di-	0.472	0.266	0.376	0.371 (0.060)	0.254	0.416	0.366	0.345 (0.048)
Tri-	0.447	0.216	0.305	0.323 (0.067)	0.346	0.32	0.437	0.368 (0.035)
Tetra-	0.206	BDL (<0.004)	0.172	0.126	0.045	0.246	0.26	0.184 (0.069)
Penta-	0.124	BDL (<0.006)	0.107	0.077	BDL (<0.006)	0.135	0.135	0.09
Hexa-	0.057	BDL (<0.002)	0.012	0.023	BDL (<0.002)	0.013	0.027	0.013
Total	1.306	0.482	0.972	0.920 (0.239)	0.645	1.13	1.225	1.000 (0.180)

SEM, Standard error of the mean; BDL, below detection limit.

### Evaluation of *B*. *poppei* permeability to rotenone

**Figure 3:**
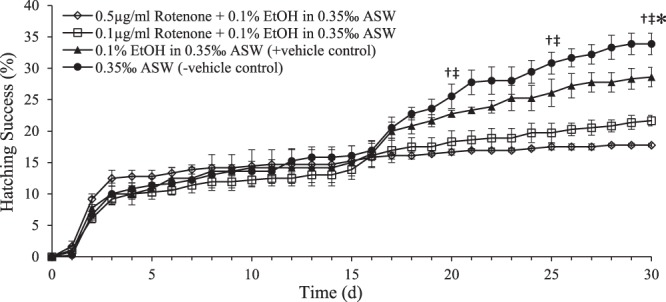
Sensitivity of *B*. *poppei* to chemical anoxia induced by rotenone exposure. Hatching success was evaluated as the percent of all individuals reaching the free-swimming nauplius stage with T = 0 as day of isolation of red embryos from sediment, and initiation of plate culturing. One way ANOVA was used to evaluate effect of treatments when 5 d, 10 d, 15 d, 20 d, 25 d or 30 d were considered as endpoints (n = 3; α = 0.05; †rotenone treatments significantly less than - vehicle control; ^‡^rotenone treatments significantly less than + vehicle control, *+vehicle control significantly less than − vehicle control).

When embryos were exposed to 0.1 µg ml^−1^ or 0.5 µg ml^−1^ rotenone at the time of introduction to aerobic incubation at 4 °C in 0.35‰ ASW, the initial hatching event (Fig. 3, day 3) was unaffected, but the second mass hatching event (Fig. 3, day 16) was blocked in a concentration-dependent manner. A Tukey’s post-hoc test demonstrates that rotenone did not significantly impact hatching success until day 18 of exposure, at which time both the 0.1 µg ml^−1^ and 0.5 µg ml^−1^rotenone treatments had significantly lower hatching success than the -vehicle control (p = 0.0417 and p = 0.0126, respectively). By day 19 the 0.5 µg ml^−1^ rotenone treatment had a significantly lower hatching success than the + vehicle control, which contained 0.1% EtOH (p = 0.0463). By day 26, both the 0.1 µg ml^−1^ and 0.5 µg ml^−1^ rotenone treatments had significantly decreased hatching success relative to the + vehicle control (p = 0.0254 and p = 0.0038, respectively). There was no significant difference between the + vehicle and –vehicle controls at any time-point (p > 0.06), nor was there a significant difference between the two rotenone treatments at any time-point (p > 0.19). Larvae found in rotenone treatments were always immotile, which indicates that they succumb shortly after hatching.

### Evaluation of *B*. *poppei* sensitivity to oxygen limitation

**Figure 4:**
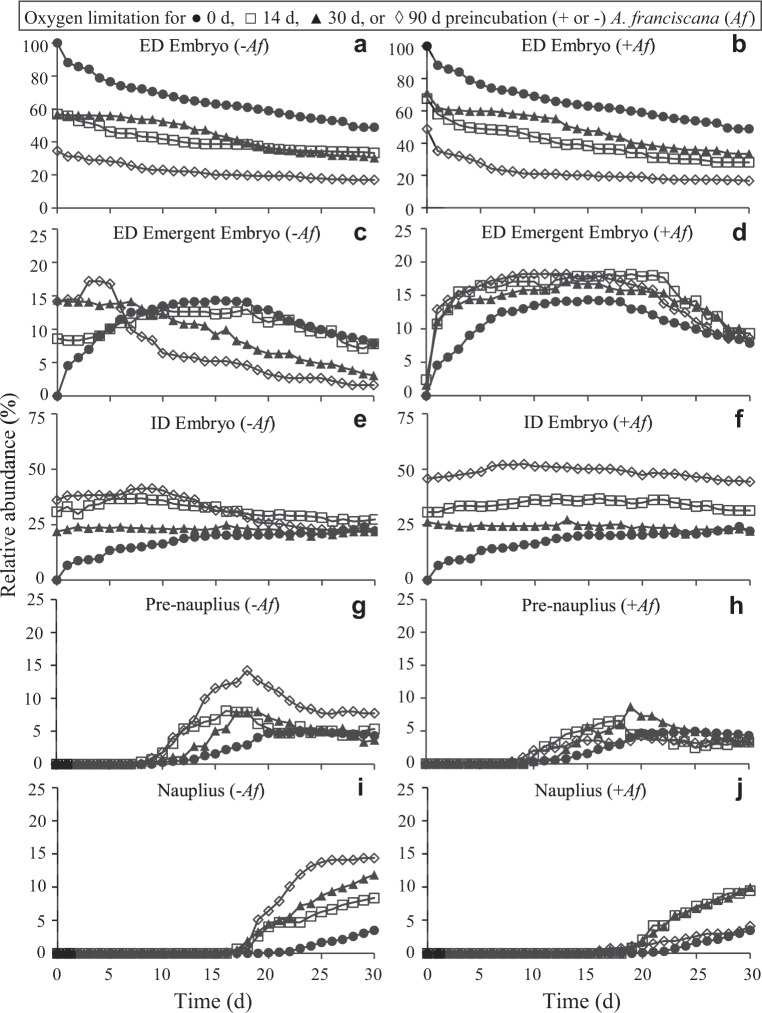
Development of *B*. *poppei* following 14 d, 30 d or 90 d of oxygen limitation produced by incubation in 0.35‰ ASW sparged with N_2_ gas in the presence (+*Af*) or absence (−*Af*) of *A*. *franciscana* embryos as oxygen scavengers. The addition of *A*. *franciscana* provided additional biomass to consume residual oxygen quickly. *Artemia franciscana* also serve to demonstrate that anoxic conditions were achieved, because embryos of this species go dormant under anoxia; 88–93% of *A*. *franciscana* in +*Af* treatments became dormant before initiation of emergence, demonstrating that anoxia was achieved in all treatments. Five categories of red *B*. *poppei* were evaluated based on development and hatching events: (**a**,**b**) early development (ED), (**c**,**d**) ED embryo emerging from cyst wall, (**e**,**f**) intermediate development (ID), (**g**,**h**) unhatched nauplius, and (**i**,**j**) free-swimming nauplius. Sum of stages shown may not equal 100, because individuals that turned white are not plotted. The same aerobic control (0 d of oxygen-limited pretreatment) data are plotted in left and right panels for comparison with each preincubation treatment condition. Data points represent mean (n = 3).

When embryos of *B*. *poppei* from lake sediments were incubated under severe oxygen limitation induced by sparging of 0.35‰ ASW media in sealed culture tubes with N_2_ gas, post-diapause development occurred, but did not progress beyond the ID stage (Fig. 4). There were significantly fewer ED embryos in the 14 d, 30 d and 90 d oxygen limited treatments than the control from day 0 to day 25 of aerobic recovery (Tukey’s HSD at individual time points, p < 0.045), after which only the 90 d oxygen limited treatment differed from the control (Tukey’s HSD at 30 d of recovery, p = 0.0033). The number of *B*. *poppei* embryos that remained in the ED stage after 14 d or 30 d of oxygen limitation (day 0 of aerobic recovery; Fig. 4a) was equal to that of the control on day 30 under aerobic conditions (student’s t-test, p = 0.1009 or p = 0.3124, respectively) (Fig. 4a). The inclusion of *Artemia franciscana* embryos as oxygen scavengers did not alter this pattern (student’s t-test p = 0.0822 or p = 0.0517) (Fig. 4b). However, the inclusion of *A*. *franciscana* did decrease the number of *B*. *poppei* embryos developing beyond the ED stage in the 90 d anoxic incubations; the number of *B*. *poppei* embryos in the ED stage at the end of 90 d under anoxia (day 0 of aerobic recovery; Fig. 4a) was significantly less than that of the control at 30 d of aerobic incubation without *A*. *franciscana* (student’s t-test, p = 0.2592), but statistically identical to the control when *A*. *franciscana* was present in the 90 d anoxic treatment (student’s t-test, p = 0.9893) (Fig. 4b).

The majority of embryos that developed under 14 d, 30 d and 90 d of oxygen limitation arrested in the ID stage of development (control vs. treatments at 0 d; Fig. 4e,f), and at numbers equivalent to that of aerobic controls after two weeks of aerobic incubation (student’s t-test, p ≥ 0.0685). Interestingly, incubation under oxygen limitation for 90 d did increase the number of embryos that reached the pre-nauplius stage by 16 d of aerobic recovery relative to the aerobic control (Tukey’s HSD, p = 0.0323) (Fig. 4g), but no oxygen limiting treatment significantly changed hatching success during aerobic recovery (one way ANOVA 30 d aerobic recovery, p = 0.1366 without *A*. *franciscana*, p = 0.4393 with *A*. *franciscana*) (Fig. 4i,j).

Up to 14.4% of *B*. *poppei* embryos incubated under low oxygen conditions emerged prematurely (ED emergent embryos) during the treatment (Fig. 4c). The number of ED emergent embryos in the 30 d and 90 d anoxic incubations was significantly greater than that of controls on day 1 of the aerobic recovery treatment (Tukey’s HSD, p = 0.0149 and p = 0.0140, respectively), and remained higher than the control throughout the aerobic recovery for the 90 d anoxic treatment group (p < 0.04). Inclusion of *A*. *franciscana* embryos as oxygen scavengers delayed the premature emergence to the day after return to normoxic conditions, but did not decrease the number of embryos that emerged prematurely (c.f. Fig. 4c,d). Control embryos also followed this premature emergence path (Fig. 4c,d), which indicates that it is a characteristic of the sediment subsamples used rather than something induced by exposure to low oxygen conditions after isolation from the sediment.

**Figure 5:**
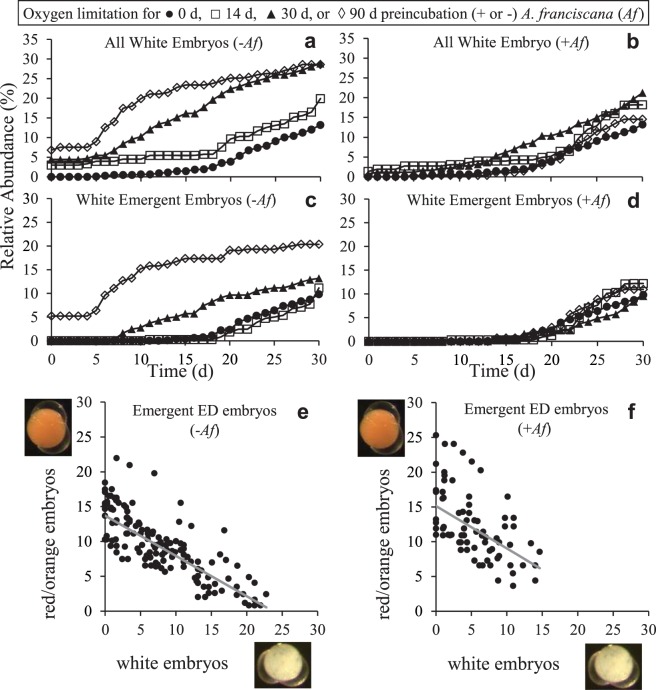
Early emergence from the cyst wall is lethal in *B*. *poppei*, and is more likely to occur when embryos are exposed to oxygen limitation early in post-diapause development. Two categories of white embryos are graphed for exposures with (+*Af*) or without (−*Af*) *A*. *franciscana* embryos in the hypoxic preincubation: (**a**,**b**) all white embryos and (**c**,**d**) white embryos emerging from cyst wall (mean values plotted, n = 3). (**g**,**h**) Regression of relative abundance of red and white emerging embryos indicates that red embryos turn into white embryos (p < 0.0001 for +*Af* and −*Af* treatment types). Reasoning for use of *Af* explained in Fig. 4.

Mortality was low during all anoxic treatments, and mortality in aerobic recovery was generally associated with premature emergence. White colour is an indicator of mortality, as evidenced by their slow decay (Reed and Covi, unpublished observations). The number of embryos that turned white during the oxygen limitation treatments was less than 7% for all treatments without *A*. *franciscana* present (Fig. 5a; day 0 of aerobic recovery), but almost no white embryos were present at the end of the oxygen limitation treatments when *A*. *franciscana* was present (Fig. 5b; day 0 of aerobic recovery). The total number of embryos that turned white by day 30 of aerobic recovery was significantly lower in oxygen limitation treatments with *A*. *franciscana* than treatments without *A*. *franciscana* (two way ANOVA; p = 0.0007 for effect of *A*. *franciscana*, p = 0.0467 for effect of anoxia duration) (c.f. Fig. 5a,b). The inclusion of *A*. *franciscana* also significantly decreased the number of embryos that emerged and turned white by day 30 of aerobic recovery (two way ANOVA; p = 0.0406 for effect of *A*. *franciscana*, p = 0.0675 for effect of anoxia duration) (c.f. Fig. 5c,d). A two way ANOVA also demonstrated that there was a significant interaction between *A*. *franciscana* presence and anoxia duration on the abundance of white embryos (p = 0.0411) and white emergent embryos (p = 0.0172) at the 30 d aerobic recovery time-point. The fate of individual embryos could not be tracked in wells, because each well contained 20–30 embryos. However, a linear regression demonstrated that a proportional increase in the presence of white (decaying) ED embryos occurs as the red ED embryos disappear during aerobic recovery (Fig. 5g,h).

Enough oxygen was present in the sealed tubes for 7–12% of *A*. *franciscana* embryos to begin the emergence process in all oxygen limitation experiments. However, the vast majority of *A*. *franciscana* embryos successfully entered a state of metabolic and developmental arrest prior to the initiation of emergence, as evidenced by subsequent hatching tests; when incubated under aerobic conditions at 22 °C under constant darkness following the 14 d, 30 d and 90 d oxygen limitation treatments at 4 °C, 70 ± 5%, 72 ± 2% and 77 ± 4%, respectively, of *A*. *franciscana* embryos successfully hatched.

### Evaluation of changes in embryo abundance during sample storage

**Figure 6:**
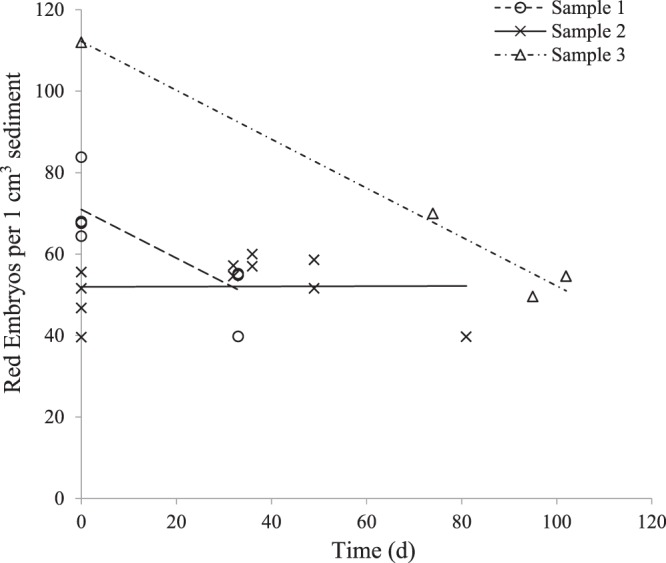
Abundance of red/orange *B*. *poppei* embryos in sediment from one lake on Barton Peninsula, King George Island, Antarctica. Day 0 is the first day each Whirl-pak® storage bag was opened since the sediment was subsampled on King George Island. Samples 1 and 2 were collected in February 2015 and stored for 16 months at 4 °C prior to opening. Sample 3 was collected in 2016 and stored for 10 months 4 °C prior to opening. Linear regressions for samples 1, 2 and 3, p = 0.0145, p = 0.9775, and p = 0.0103, respectively.

When lake sediments containing embryos of *B*. *poppei* were stored at 4 °C in sealed Whirl-pak® bags with secondary containment in black plastic containers to block light exposure, total embryo abundance decreased significantly over time for two of three sediment samples that were tracked quantitatively (Fig. 6). As with the oxygen-limiting chambers, it was not possible to measure oxygen tension or light exposure in the bag without introducing oxygen and light to the sample.

## Discussion

Zooplankton of coastal and inland waters produce an “egg bank” that can provide resilience for a population by maintaining the genetic diversity required to deal with environmental change^2,36,49^, but the embryos that make up these sediment egg banks are now threatened by anthropogenic activities. Persistent organic pollutants could pose a particularly serious threat to the egg bank if they were able to penetrate the protective cyst wall of the dormant embryo. In order to evaluate the potential for lipophilic POPs to penetrate and passively accumulate in dormant embryos of Antarctic lacustrine zooplankton, we evaluated the effect of anoxia on post-diapause development in the calanoid copepod, *B*. *poppei*, and then used chemical anoxia induced by rotenone exposure as a reporter for permeability of these embryos to moderately lipophilic chemicals. We succeeded in demonstrating (1) that post-diapause *B*. *poppei* develop under severe hypoxia, (2) that hatching does not occur under severe hypoxia and (3) that rotenone passively penetrates the cyst wall and blocks hatching in a manner consistent with severe hypoxia. From this, we infer that long-term storage of *B*. *poppei* embryos requires deposition in anoxic sediments, and that embryos in these sediments will passively absorb moderately lipophilic chemicals. A chemical analysis of sediments from Antarctic lakes with *B*. *poppei* populations indicates that legacy POPs with predicted octanol-water partition coefficients similar to rotenone (logP = 4.01) are present and likely to accumulate in dormant *B*. *poppei*.

Previous studies on *Boeckella* spp. describe the effects of physical variables on generation time, embryo development and hatching success^50–52^, but none provide a clear description of early developmental events that can be used to characterize the impacts of environmental variables on development. To the best of our knowledge, the present work is the first to fully describe encysted post-diapause development, emergence and hatching in a member of the genus, *Boeckella*, in a manner that can be used to quantitatively evaluate the impacts of environmental variables on these events. Emergence and hatching both depend on the osmotic expansion of a space between a thin hatching membrane and the fully formed nauplius larva (Fig. 1). Two outer layers matching the dimensions of the diapause embryo are shed sequentially during expansion of this space, and the hatching membrane eventually ruptures as a consequence of burst swimming by the enclosed nauplius (Video S1). This sequence of events described previously for the brine shrimp, *A*. *franciscana*^10,53^, is very similar to that of *B*. *poppei*, but the proportional expansion of the space between the hatching membrane and nauplius larva is much larger in *B*. *poppei*. Emergence and hatching in *Artemia* spp. appear to depend on the action of a chitinase working in concert with osmotic swelling^54,55^. The role of chitinases in emergence and hatching of copepods remains unexplored.

As is the case for post-diapause embryos of *A*. *franciscana*^10^, development and hatching in embryos of *B*. *poppei* from Antarctic lake sediments are independent processes coordinated as part of a larger developmental program which is sensitive to oxygen, but not salinity or light. Neither light, nor the variation of salinity between 0.035‰ and 4.5‰, alter the timing of emergence events or hatching success in embryos of *B*. *poppei* that are separated from lake sediments (Fig. 2). By contrast, exposure to severe hypoxia blocks hatching without stopping development (Fig. 4). The lack of sensitivity to moderate osmotic challenge is not surprising, given that members of the *Boeckella* genus inhabit freshwater, brackish water and hypersaline lakes^52,56,57^. The lack of effect of photo-cues is also unsurprising, given that light penetration to the sediment surface is highly variable in lakes where this species occurs^58^, and that hatching in other calanoid copepods does not appear to depend on light cues^59,60^.

Premature emergence in *B*. *poppei* occurs in a proportion of the population of embryos in Antarctic lake sediments stored at 4 °C (Fig. 4), and is lethal (Fig. 5). This premature emergence could result from a loss of osmoregulatory control, the inappropriate activation of endogenous chitinases or the exogenous action of microbial chitinases. Importantly, the number of embryos that emerge prematurely varies among sediment sub-samples, but does not change with prolonged exposure to anoxic conditions in sterile artificial freshwater (Fig. 4). This indicates that the events responsible for setting the lethal early emergence path are irreversible, and occur prior to isolation of embryos from the sediment. It is possible that variability in the decrease of embryo abundance after opening of sediment sample bags (Fig. 6) is caused by differences in the degree of premature emergence that occurs once oxygen is introduced to a sample. Variability in activation of development and hatching success may depend on sediment heterogeneity with respect to chemical composition, microbial communities, or a combination of the two. It is also possible that there are both diapause and quiescent embryos present in the sediment, and that heterogeneity in activation upon opening of sample bags results from variation in the proportion of diapause to quiescent subitaneous eggs. Both types of eggs are produced by *Boeckella triarticulata*, and the structural differences between them are impossible to see in live embryos with light microscopy^61^. Regardless of the reason it occurs, the possibility that embryos of *B*. *poppei* survive as long as two centuries in a native sediment egg bank, as reported by Jiang, Zhao, Xu, Wang, He and Cai^5^, makes investigation of the variable responsible for premature emergence worthy of future investigation.

An evaluation of the impacts of oxygen limitation demonstrates that embryos of *B*. *poppei* found in Antarctic lake sediments require an anoxic environment in order to remain dormant. For a broad diversity of zooplankton species, the development of dormant embryos is activated by oxygenation of sediments in which the embryos are deposited^62^. This is the case for *B*. *poppei*; hatching of *B*. *poppei* found in Antarctic lake sediments is biphasic, and prolonged incubation under aerobic conditions appears to be the cue required for most embryos to initiate development (Fig. 2). Once initiated, development in *B*. *poppei* continues under severely hypoxic conditions, but hatching is blocked until aerobic conditions are restored (Fig. 4). This response matches that of the calanoid copepods, *Acartia tonsa*, *Acartia bifilosa*, *Labidocera aestiva* and *Eurytemora affinis* when they are incubated in hypoxic chambers similar to those used in the present study^60,63–65^. Oxygen concentrations below 0.2 ml O_2_ L^−1^ are required to maintain dormancy in post-diapause embryos of these calanoid copepods^60,63–65^. It is improbable that sediment in the shallow oligotrophic lakes that *B*. *poppei* were collected from for the present study would maintain oxygen concentrations this low for centuries, which raises questions about how embryonic dormancy could be maintained for almost two centuries in *B*. *poppei*, as reported by Jiang, Zhao, Xu, Wang, He and Cai^5^.

An evaluation of the impacts of chemical anoxia on embryos of *B*. *poppei* from Antarctic lake sediments demonstrates that the cyst wall surrounding these embryos is permeable to moderately lipophilic chemicals. The physiological effects of chemical anoxia induced by exposure to rotenone should mimic those of true anoxia. In the case of zooplankton embryos that exhibit anoxia-induced quiescence, chemical anoxia should decrease hatching success without increasing embryo mortality. This is exactly what occurs during rotenone exposure for dechorionated post-diapause embryos of the brine shrimp, *A*. *franciscana*^48^. The present study demonstrates that unmodified *B*. *poppei* embryos isolated from Antarctic lake sediments fail to hatch in the presence of rotenone (Fig. 3). Rotenone does not block the embryos from hatching during the first week of exposure, even though the concentrations of rotenone used caused 100% mortality for nauplii after hatching (Fig. 3). This delayed effect of rotenone on hatching indicates that the cyst wall provides short-term protection from compounds like rotenone. Rotenone is a moderately lipophilic compound with a logP value in the range reported for PCBs and their metabolites^66^. It is, therefore, reasonable to suggest that chemicals like persistent organochlorines will passively partition to embryos of *B*. *poppei* in Antarctic sediments.

While it is well known that a diverse array of POPs are present in Antarctic food webs^30,32,67^, we are aware of no previous studies that evaluate the presence or impact of these chemicals on maritime lacustrine systems in Antarctica. Relatively high levels of PCBs are present in terrestrial soil, lichen, marine sediment, marine fish, and seabirds on, or just off shore of, Barton Peninsula, King George Island^26,27^. An evaluation of the collection sites used by Park, Lee, Kim, Kim and Lim^27^ demonstrates that 32 PCB congeners, including 12 dioxin-like PCBs, are present in terrestrial soil and lichen found in catchments of freshwater lakes. The most common PCB homologues in terrestrial samples on Barton Peninsula are Di and Tri-PCBs, while Hepta, Octa, Nona and Deca-PCBs are relatively rare^27^. This is similar to the profile of PCBs in lake sediments on Barton Peninsula that were identified in the present study (Table 1). It is important to note, however, that the concentrations of PCBs in lake sediments are one to two orders of magnitude greater than Park, Lee, Kim, Kim and Lim^27^ reported for terrestrial soil. This demonstrates that lakes in the maritime Antarctic act as sinks for legacy POPs. Recent data demonstrate that levels of POPs in two species of icefish are similar across a large region near the Antarctic Peninsula, which includes the South Shetland Islands^32^. Future research should investigate whether this trend holds true for sediment or zooplankton in coastal lakes across the same broad maritime region.

The demonstrated susceptibility of lacustrine zooplankton embryos to moderately lipophilic chemicals should raise alarm, because it places potentially ancient, and poorly studied, populations at risk. It is highly likely that dormant embryos deposited in Antarctic lake sediments over the last half century contain a diverse array of POPs, because these lipophilic chemicals bioaccumulate in active lacustrine zooplankton^68^ and appear in dormant embryos of species from contaminated waters^10^. The present study suggests that embryos of the copepod, *B*. *poppei*, deposited in lake sediment more than a century before POPs appeared in Antarctica will contain these chemicals, because passive partitioning of moderately lipophilic compound occurs across the embryonic cyst wall. Copepods are not the only zooplankton group of concern either; pesticides with LogP values similar to that of rotenone penetrate the protective ephippium surrounding dormant cladoceran embryos, and negatively impact recruitment from egg banks^9,12^. With this in mind, it is tempting to ask what anthropogenic chemicals are present in dormant zooplankton embryos today, and what effect these chemicals might have on recruitment from egg banks in Antarctic lakes. Antarctic lacustrine zooplankton also play a role in controlling microbial and ciliate community structure^57,69^, so questions about how adverse effects on dormant zooplankton will affect eutrophication in warming lakes are also tempting to ask. However, assaying these rare embryos for diverse chemical species would be challenging at best, and attempting to evaluate the impacts of chemicals on community structure would be exceedingly difficult. Perhaps a more productive question to ask is whether a passive system can be deployed in these lakes to bind lipophilic chemicals before they have a chance to partition to the zooplankton embryo.

## Methods

### Chemicals

All solutions were prepared using ultrapure deionized water (resistivity ≥ 18 MΩ cm at 25 °C). Food-grade table sugar was used to prepare sucrose solutions for density-dependent isolation of dormant zooplankton from sediments. Instant Ocean® artificial sea salts (Spectrum Brands, Blacksburg, VA, USA) were used to prepare artificial seawater (ASW). All other chemicals used in the isolation, preincubation and culturing of zooplankton were of ACS grade or higher.

### Preparation of solutions for culturing of zooplankton

Stock solutions of 20‰ or 35‰ ASW were prepared, and salinity was determined with a refractometer (Vee Gee Scientific, Kirkland, WA, USA) at room temperature (approximately 22 °C) before dilution to generate working solutions of 0.35‰, 1.75‰, 3.5‰ or 4.5‰. All stock and working solutions were sterilized by vacuum filtration through a 0.2 μm polyethylsulfone (PES) filter and stored at 4 °C in sterile glass bottles until use.

### Origin and preparation of zooplankton

Encysted embryos of the brine shrimp, *A*. *franciscana* (Great Salt Lake population), were purchased in the dehydrated state from Sanders Brine Shrimp (Ogden, UT, USA) in 2012, and stored in an airtight canning jars at −20 °C. Embryos were hydrated in 0.25 M NaCl for 4 h at 0 °C. Hydrated embryos were decanted into a Durawipe® cloth (Chicopee, Charlotte, NC, USA), and rinsed with ASW of a salinity matched to the experimental treatment. Prior to weighing of embryos, excess ASW was drawn out by blotting the sides of the Durawipe® cloth briefly with Kimwipes® (Kimberly-Clark, Neenah, WI, USA).

Sediment samples containing embryos of the Antarctic copepod, *B*. *poppei*, were collected from one maritime lake on Barton Peninsula, King George Island, Antarctica, (62.239869°S, 58.744733°W) in February 2015 and January 2016, and shipped at 4 °C to the University of North Carolina at Wilmington via the United States Antarctic Program (USAP) peninsula logistics. The maximum temperature recorded for all samples during shipment was 9 °C. Unless otherwise stated, sediment samples were stored at 4 °C and shielded from light until use. All procedures for processing of sediment samples were performed under dim red light provided by a 25 W 120 V incandescent bulb with opaque coating (Feit Electric Company, Pico Rivera, CA) in an environmental control room that maintained air temperature between 2 °C and 4 °C.

Dormant embryos were separated from lake sediment using a modified version of the sugar floatation method described by Briski *et al*.^70^. In brief, 5 ml of sediment was added to 45 ml of 80% sucrose, mixed by gentle inversion and centrifuged for 2 min at 1000 rcf with centrifuge chamber temperature maintained between 0 °C and 4 °C. The supernatant, which contained the zooplankton embryos, was then poured over a 63 µm stainless steel sieve. In order to remove residual sucrose, embryos were rinsed on the sieve with 500 ml of the solution used for culturing or preincubation. Embryos were then transferred from the sieve to a petri dish for visual examination at 100X magnification under a Nikon SMZ745T dissecting microscope with G-AL 2X auxiliary lens (Nikon, Sterling Heights, MI, USA). Embryos were characterized by colour, internal structure and condition of the cyst wall. Unless otherwise stated, all embryos selected for experiments were red or orange in colour, possessed an unruptured cyst wall and were in an early stage of embryonic development that lacked any clear evidence of bilateral symmetry or segmentation at 100X magnification.

### Evaluation of *B. poppei* salinity impact on hatching

Embryos isolated from sediment that was collected in February 2015 were used to test the impact of mild salinity change on development and hatching in July 2016. Three experimental replicates were generated by conducting three separate embryo preparations using sediment from the same Whirl-pak® storage bag (Nasco, Fort Atkinson, WI, USA), which had remained unopened since collection in 2015. Immediately after isolation from sediment, 20–30 embryos were transferred to each well of a sterile Cellstar® 12-well polystyrene culture plate (Greiner bio-one, Monroe, NC, USA) containing 1 ml of sterile 0.35‰, 1.75‰, 3.5‰ or 4.5‰ ASW per well. Four wells of a 12-well plate containing 20–30 embryos each (130–167 embryos in total) were used for each treatment. Embryos in 12-well culture plates with clear plastic lids were incubated in an environmental chamber under constant light provided by four ‘full-spectrum’ 32 W Ecolux-with-starcoat General Electric florescent bulbs (General Electric Co., Fairfield, CT, USA). Development, emergence and hatching were recorded every 12 h for 30 days. Illuminance and air temperature were monitored once per min with a HOBO UA-002-64 data logger (Onset Computer Corporation, Bourne, MA, USA) adjacent to the culture plates. During a representative week, the air temperature was 3.49 ± 0.01 °C, and illuminance was 830.6 ± 0.4 lux. Volume loss from wells containing 0.35‰ ASW was approximately 0.5% per week under these conditions, and there was no visible difference in water level among treatment types. No attempt was made to compensate for volume loss by evaporation.

### Evaluation of light requirement for hatching of *B*. *poppei*

To determine the effect of light exposure on the timing or extent of hatching for a population of *B*. *poppei*, constant light and constant darkness treatments were compared. With the exception of light exposure, the protocol followed that outlined for the test of salinity and an additional dilution of ASW to 0.035‰ was added. To investigate the effect of light restriction on hatching across a permissive range of salinities, one set of embryos in 12-well plates was incubated under constant light while a second set of identical plates were incubated under constant darkness with the exceptions. Exposure to light from a Nikon NI-150 halogen light with dual fiber optic cables during daily observation was estimated by setting up the dissecting microscope for observation under the dissecting microscope and replacing the culture plate with a HOBO UA-002-64 data logger.

### Analysis of PCB content in Antarctic lake sediments

Samples were treated, extracted, and analyzed according to the US Environmental Protection Agency (USEPA) method 1668A^71^. Samples of approximately 50 g of soil were spiked with ^13^C-labeled PCB internal standards (1, 3, 4, 15, 19, 37, 54, 77, 81, 104, 105, 114, 118, 123, 126, 155, 156, 157, 167, 169, 188, 189, 202, 205, 206, 207 and 209), and extracted for 16 h using a hot Soxhlet manifold with 300 mL of toluene. The extract was collected and concentrated to approximately 2 mL using a rotary evaporator, and further purified using a column filled with silica gel. The column consisted of (from bottom to top) quartz glass wool, 0.9 g of silica gel, 3 g of 2% KOH silica gel, 0.9 g of silica gel, 4.5 g of 44% H_2_SO_4_ silica gel, 6 g of 22% H_2_SO_4_ silica gel, 0.9 g of silica gel, 3 g of 10% AgNO_3_ silica gel, and 6 g of sodium sulfate. The column was washed with 50 mL of hexane immediately prior to use. The extract was passed through the column and eluted with 120 mL of hexane. The eluate was concentrated to 5 mL using a rotary evaporator and then reduced to 0.5 mL under a gentle stream of nitrogen gas. Finally, 1.2 ng of ^13^C12-labeled injection standards (9 L, 52 L, 101 L, 138 L and 194 L) were added as internal standards prior to analysis. Dioxin-like PCB congeners were identified and quantified by high-resolution gas chromatography/high-resolution mass spectrometry (HRGC/HRMS) on a DFS mass spectrometer (Thermo Fisher Scientific, Bremen, Germany) using USEPA method 1668A^71^ for PCB congeners. The mass spectrometer was operated in electron impact mode (36 eV) with helium gas as a carrier. The GC column was a DB-5MS fused silica column (60 m × 0.32 mm i.d., 0.20-µm film thickness). The column oven temperature was programmed to increase at a rate of 20 °C/min from an initial temperature of 120 °C (3-min hold) to a temperature of 220 °C (5-min hold), then at 4 °C/min to 260 °C (17-min hold). The injector, transfer line, and ion source were all at a temperature of 260 °C. The identification of dioxin-like PCBs was performed based on the retention times of the ^13^C-labeled standard and isotope ratios M/(M + 2) or (M + 2)/(M + 4). The concentrations of native analytes with corresponding ^13^C-labeled surrogate standards were calculated using the isotope dilution method of quantification based on EPA method 1668A^71^. The average recovery of internal standards was 89 ± 27%. The limits of detection for selected major PCBs found in samples were 0.001–0.041 pg/g dry weight.

### Evaluation of *B. poppei* sensitivity to rotenone

Embryos isolated from sediment that was collected in February 2015 were used to test the impact of rotenone on hatching success in April 2016 according to the same protocol as tests of sensitivity to salinity, but with the following modifications. The maximum number of embryos per well of the 12-well plates was increased from 30 to 35. Rotenone was dissolved in 100% ethanol (EtOH) on the day of use and added to the sterile 0.35‰ ASW just prior to the introduction of embryos. The final concentration of the EtOH vehicle was 1% for all rotenone treatments, and the effect of EtOH on *B*. *poppei* was assessed with a control that contained 1% EtOH in 0.35‰ ASW.

### Evaluation of *B*. *poppei* sensitivity to oxygen limitation

Embryos isolated from sediment collected in January 2016 were used to test the impact of oxygen limitation on development and hatching in July 2017. Within 2.5 h of isolation from lake sediment, 105 embryos were transferred by micro-pipette into an airtight culture tube with a butyl rubber plug-style cap secured with a plastic screw lid (Chemglass Life Sciences, Vineland, NJ, USA) that contained 10 ml of 0.35‰ ASW. Prior to the addition of the embryos, the ASW was sparged for 20 min in the culture tube by bubbling with 99.99% nitrogen (N_2_). To prevent back diffusion of oxygen into the tube, the N_2_ gas was passed through the rubber cap with a 21-gauge needle, and gas was vented through a 20-gauge needle to prevent pressurizing of the tube. Approximately 20 µl of 0.35‰ ASW equilibrated with room air was added with the embryos. In order to remove oxygen introduced during embryo transfer, N_2_ gas was passed into the headspace above the incubation medium for an additional 10 min after the introduction of the embryos. The needles were withdrawn from the self-sealing rubber cap when sparging with N_2_ was complete. To produce anoxic conditions, the same procedure was repeated with embryos from the same sediment preparation, but 0.3 g of hydrated *A*. *franciscana* embryos were added to remove residual oxygen. Post-diapause embryos *A*. *franciscana* will consume oxygen in closed vessels, and will enter a reversible state of dormancy when oxygen is depleted^41,46,72^. The efficacy of the oxygen removal was then assessed by evaluating whether the *A*. *franciscana* began the hatching process during the experiment.

The culture tubes containing *B*. *poppei*, or *B*. *poppei* and *A*. *franciscana*, remained sealed for 14, 30 or 90 days, after which *B*. *poppei* were transferred to 12-well culture plates containing 0.35‰ ASW. To mimic conditions during the austral summer, a 19:5 light:dark cycle was maintained during incubation in sealed tubes and culturing in plates. As an aerobic control, embryos from the same sediment sample were transferred directly to the 12-well culture plates on the day that the hypoxic or anoxic incubations were initiated. Development, emergence and hatching were recorded daily for a period of 30-days with day 0 defined as the day that embryos were placed in 12-well culture plates. To compensate for volume loss during the 30-day aerobic period, 10 µl of ultrapure water (1% of starting ASW volume) was added at day 14 and day 28.

### Assessment of development in *A. franciscana* after anoxic treatments

*Artemia franciscana* embryos were examined at 75X magnification immediately after anoxic treatments were terminated in order to determine if emergence or hatching had occurred in the sealed tubes. Embryos that demonstrated no signs of emergence were transferred to sterile 12-well culture plates containing 1 ml of sterile 20‰ ASW per well; 32–40 embryos were placed in each well, providing a total of 384–480 embryos from each anoxic treatment. Culture plates were incubated at 22 ± 0.5 °C under constant darkness, and emergence and hatching were recorded once per day for seven days to assess viability. All hatched larvae were removed on day four of the hatching test, because food was not provided to support further development. Stage identification for *A*. *franciscana* was based on Neumeyer, Gerlach, Ruggiero and Covi^10^.

### Data analysis

A true replicate was considered to be an individual embryo, and the data were plotted as a percent calculated from observations of greater than 100 embryos to assess the response of the population to treatment conditions. Experimental replication included separate subsampling of stored lake sediment containing dormant *B*. *poppei*. Relative abundance for developmental stages of *B*. *poppei* are either plotted individually or as a mean; a mean was calculated only when the experimental replicates demonstrated similar trends in plots of individual replicates. When a mean of three or more individual replicates was plotted, mean values were compared with statistical analyses conducted in JMP Pro 12.01.1 (SAS Institute, Cary, NC, USA). All data are available in a single supplementary spreadsheet file (Table S1).

## Electronic supplementary material

Supplementary Video S1

Supplementary Table S1

## Data Availability

All data generated or analyze during this study are included in this published article and its Supplementary Information files (Supplementary Dataset Table S1).
